# Expression of Concern: Menstrual hygiene practices among high school girls in urban areas in Northeastern Ethiopia: A neglected issue in water, sanitation, and hygiene research

**DOI:** 10.1371/journal.pone.0329125

**Published:** 2025-07-29

**Authors:** The PLOS One Editors

The *PLOS One* Editors issue an Expression of Concern to alert readers that this article [1] was identified as one of a series of submissions for which we have concerns about authorship and adherence to the journal’s first and second publication criteria. We regret that these issues were not addressed prior to the article’s publication.
