# Supplementary material for: Menstrual hygiene practices among high school girls in urban areas in Northeastern Ethiopia: A neglected issue in water, sanitation, and hygiene research
Source: PLoS One. 2021 Jun 9;16(6):e0248825. doi: 10.1371/journal.pone.0248825 (PMC8189485; doi:10.1371/journal.pone.0248825)
Supplement: S1 Appendix — Survey of menstrual hygiene practices among high school girls in urban areas in northeastern Ethiopia: A neglected issue in water, sanitation, and hygiene research. (DOCX) [file pone.0248825.s001.docx]

S1 Appendix. Questionnaires in English version

| **Socio-demographic and Economic data** | | | |
| --- | --- | --- | --- |
| No | Questions | Possible answer | skip |
|  | School type? | 1. Public 2. Private |  |
|  | Your Age (years)? |  |  |
|  | Your Grade | 1. 9^th^ 2. 10^th^ |  |
|  | Your religion? | 1. Orthodox 2. Muslim 3. Protestant 4. Catholic 5. Others (specify) |  |
|  | Place of residence? | 1. Urban 2. Rural |  |
|  | Your marital status | 1. Single 2. Married 3. Divorced 4. Widowed 5. Not applicable |  |
|  | With whom do you live? | 1. Both parents 2. Mother only 3. Father Only 4. Relatives 5. Alone 6. Others (specify) ---- |  |
|  | What is your Mother’s educational status? | 1. Illiterate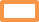 2. Read and write 3. Primary 4. Secondary 5. College and above |  |
|  | What is your Father’s educational status? | 1. Illiterate 2. Read and write 3. Primary 4. Secondary 5. College and above |  |
|  | What is your mother’s occupational status? | 1. Housewife 2. Merchant 3. Private organization employee 4. Governmental employee 5. Daily laborer 6. Others (specify) ----- |  |
|  | What is the occupational status of your father? | 1. Government Employee 2. Private Employee 3. Daily laborer 4. Self-Employee 5. Farmer 6. Others (specify)------- |  |
|  | Your family’s monthly income (ETB) | ……………………… |  |
|  | Do you earn permanent pocket money from  the family | 1. Yes 2. No |  |
| **Obstetric and gynecological related characteristics** | | | |
|  | Your age at menarche (in years) | -------------------- |  |
|  | Regularity of menses for the last six consecutive menstrual cycles? | 1. Regular 2. Irregular |  |
|  | How many days do your menstrual bleeding stayed? /Duration of menses flow/ | 1. Less than 2 days 2. 3 to 7 days 3. More than 7 days |  |
|  | Severe pain during menstruation? /3 cycle/ | 1. Yes 2. No |  |
| **Knowledge and awareness about menstruation** | | | |
|  | Did you hear about menstruation before attaining menarche? | 1. Yes 2. No | If no,  Skip 30 |
|  | What is menstruation? | 1. Physiological process 2. Pathological process 3. Curse from god 4. Others (specify) ------- 5. Don’t know |  |
|  | What is the cause of menstruation? | 1. Hormones 2. Curse of god 3. Caused by disease 4. Others (specify) ------- 5. Don’t know |  |
|  | Where is the source of menstrual blood? | 1. Uterus 2. Vagina 3. Bladder 4. Abdomen 5. Others (specify) ------- 6. Don’t know |  |
|  | Which one is the correct intermenstrual interval? | 1. Less than 21 days 2. 21 to 35 days 3. More than 35 days 4. Don’t know |  |
|  | How long does the bleeding usually take place during menstruation, in a healthy girl? | 1. Less than 2 days 2. 3 to 7 days 3. More than 7 days 4. Don’t know |  |
|  | Do you learn about menstruation and menstrual hygiene in the school? | 1. Yes 2. No |  |
|  | Do you know that there is foul-smelling during menstruation? | 1. Yes 2. No |  |
|  | Do you know that menstrual blood is unhygienic | 1. Yes 2. No |  |
|  | Do you know that poor hygiene during menstruation predispose to infection | 1. Yes 2. No |  |
|  | Do you know that personal hygiene during menstruation has a place in the prevention of menstrual pain | 1. Yes 2. No |  |
|  | Do you know that Menstruation is not a lifelong process? | 1. Yes 2. No |  |
| **Source of information and communication about menstruation** | | | |
|  | What was your source of awareness about menarche? | 1. Mother 2. School (media, teacher,) 3. Friend 4. Elder sisters 5. Television 6. Health professional 7. Internet 8. Father 9. Others (specify) ------- |  |
|  | Do you discuss about menstrual hygiene with your friends? | 1. Yes 2. No |  |
|  | Is there open communication about menstruation with your family? | 1. Yes 2. No | If no skip 33 |
|  | With whom do you frequently communicate? | 1. Mother 2. Father 3. Sister 4. Another member of the family |  |
|  | Why is there no communication about menstruation in your family? | 1. It is shameful 2. It is kept as a secret 3. All 4. Other (specify)--------- |  |
|  | Do you communicate about menstruation with your teachers? | 1. Yes 2. No |  |
| **WASH-related factors** | | | |
|  | How often is the water source functional per week? /by the last month/ | 1. 5-7 days per week 2. 2-4 days per week 3. Fewer than 2 days per week |  |
|  | Are there separate latrines for boys and girls? (observational) | 1. Yes 2. No |  |
|  | Functionality of toilets? Accessible for disabled? (observational) | 1. Functional 2. Partially functional 3. Not functional 4. Don’t know | If the answer is 4 skip Q 39,40 |
|  | Comments on: cleanliness? Smell? Of the toilet? (observational) | 1. Clean 2. Somewhat clean 3. Not clean |  |
|  | Light inside the toilets? (observational) | 1. Light 2. Somewhat dark 3. Dark |  |
|  | When can students use latrines? | 1. During Breaks only? 2. Anytime? |  |
|  | Does the school have any private disposal waste bins inside the latrines for disposable napkins(observational) | 1. Yes 2. No |  |
|  | Are girls’ individual toilet compartments lockable from the inside? (observational) | 1. All 2. Some 3. None 4. Not applicable (no school sanitation facilities) |  |
|  | Do girls’ individual toilet compartments contain anal cleansing materials (water, toilet tissue)? (observational) | 1. All 2. Some 3. None 4. Not applicable (no school sanitation facilities) |  |
|  | Does the school have hand-washing facilities? (observational) | 1. Yes 2. No |  |
|  | Water available currently? (observational) | 1. Yes 2. No |  |
|  | Is soap or ash available for personal hygiene? (observational) | 1. Yes 2. No |  |
|  | Are sanitary napkins available for girls at the school in an emergency/accident situation? (observational) | 1. Yes 2. No |  |
|  | Does the school have any private facilities for girls to bathe/wash (such as a tap and basin inside a lockable toilet stall)? (observational) | 1. Yes 2. No |  |
|  | Is there an isolated place to change the sanitary pad? (observational) | 1. Yes 2. No |  |
| **The practice of menstruation hygiene** | | | |
|  | Do you use absorbent materials during menstruation? | 1. Yes 2. No | If no skip 53 |
|  | What materials do you use during the last 6-month menstrual period? | 1. Commercially made sanitary pads 2. Homemade absorbents 3. Others (specify) ------- |  |
|  | What materials do you use for washing your reusable absorbent(s)? | 1. With soap and water 2. With water, only 3. Others (specify) -- |  |
|  | Where do you put/keep your reusable absorbent(s) after washing for drying? | 1. In the sunlight 2. In the shade 3. Others (specify) ------- |  |
|  | How often do you change absorbent material per day? | 1. Once 2. Twice 3. Three times 4. More than three times |  |
|  | Do you clean your genitalia during menstruation? | 1. Yes 2. No |  |
|  | What do you use for cleaning your external genitalia? | 1. Soap and water 2. Water only 3. Plain paper 4. Others (specify) --- |  |
|  | Do you take a shower daily during menstruation? | 1. Yes 2. No |  |
|  | What materials do you use for showering during menstruation? | 1. With soap and water 2. With water, only 3. Others (specify) |  |
|  | Where do you dispose of menstrual material used at school? | 1. Open field 2. Latrine 3. Waste bins 4. Others (specify) ------ |  |
|  | Disposes of the pads by wrapping with paper | 1. Yes 2. No |  |
| **Sanitary pad** | | | |
|  | What is your reason for not using a commercially made sanitary pad? | 1. Cost 2. Not available 3. Difficulty in disposal 4. No knowledge 5. Shyness 6. Others (specify) | More than one answer is possible |
|  | Do you ask for money from your family for buying sanitary pads? | 1. Yes 2. No |  |
|  | From whom do you get money for buying sanitary pads? | 1. Mother 2. Father 3. Elder sister 4. Brother 5. Other |  |
| **Menstruation on girls’ academic performance** | | | |
|  | How many days do you miss school because of the period during the previous semester? |  |  |
|  | I miss school during my period because I am afraid of staining my clothes. | 1. Yes 2. No |  |
|  | I miss school during my period because I am afraid of others making fun of me. | 1. Yes 2. No |  |
|  | I miss school during my period because periods can cause pain. | 1. Yes 2. No |  |
|  | I miss school during my period because periods can make me feel uncomfortable or tired. | 1. Yes 2. No |  |
|  | I miss school during my period because there isn’t anywhere for girls to wash at school. | 1. Yes 2. No |  |
|  | I miss school during my period because there is nowhere to dispose of sanitary products. | 1. Yes 2. No |  |
|  | I miss school during my period because I do not have sanitary pads. | 1. Yes 2. No |  |
|  | I miss school during my period because there isn’t anywhere for girls to change pad at school | 1. Yes 2. No |  |
