# Supplementary material for: Menstrual hygiene practices among high school girls in urban areas in Northeastern Ethiopia: A neglected issue in water, sanitation, and hygiene research
Source: PLoS One. 2021 Jun 9;16(6):e0248825. doi: 10.1371/journal.pone.0248825 (PMC8189485; doi:10.1371/journal.pone.0248825)
Supplement: S2 Appendix — Survey of menstrual hygiene practices among high school girls in urban areas in northeastern Ethiopia: A neglected issue in water, sanitation, and hygiene research. (DOCX) [file pone.0248825.s002.docx]

S2 Appendix. የአማርኛ ቃለመጠይቅ

ኮድ ----------- የትምህርት ቤቱ ስም ----------------------------------------------------

ከቃለ ምልልሱ በኋላ የተሟላና ያልተሟላ መሆኑን ለመለየት በሳጥኑ ውስጥ ምልክት ያድርጉ

የተሟላ ያልተሟላ

| **የስነ-ህዝብ እና የኢኮኖሚ መረጃ** | | | | |  |
| --- | --- | --- | --- | --- | --- |
| **ተ.ቁ** | **ጥያቄ** | | **መልስ** | **ዝለል** |  |
|  | የት/ቤቱ አይነት | | 1. የመንግስት 2. የግል |  |  |
|  | እድሜ | |  |  |  |
|  | የክፍል ደረጃ | | 1. 9^ኛ^ 2. 10^ኛ^ |  |  |
|  | ሃይማኖት | | 1. ኦርቶዶክስ 4. ካቶሊክ  2. ሙስሊም 5. ሌላ (ይገለፅ) --  3. ፕሮቴስታንት |  |  |
|  | የመኖሪያ ቦታ | | 1. ከተማ 2. ገጠር |  |  |
|  | የጋብቻ ሁኔታ | | 1. ያላገባች 4. የሞተባት  2. ያገባች 5. የማይመለከተው  3. የፈታች |  |  |
|  | ከማን ጋር ነው የምትኖሪው? | | 1. በጋራ /እናት፣አባትጋር/ 4. ዘመድ  2. እናት ብቻ 5. ብቻየን  3. አባት ብቻ 6. ሌላ (ይገለፅ) --- |  |  |
|  | የእናት የትምህርት ደረጃ | | 1. ያልተማረች 4. ሁለተኛ ደረጃ  2. ማንበብና መጻፍ 5. ኮሌጅ/ዩኒቨርስቲ  3. የመጀመሪያ ደረጃ |  |  |
|  | የአባት የትምህርት ደረጃ | | 1. ያልተማረ 4. ሁለተኛ ደረጃ  2. ማንበብና መጻፍ 5. ኮሌጅ/ዩኒቨርስቲ  3. የመጀመሪያ ደረጃ |  |  |
|  | የእናት የስራ ሁኔታ | | 1. የቤት እመቤት 4.የመንግስት ሰራተኛ  2. ነጋዴ 5. የቀን ሰራተኛ  3. የግል ድርጅት ሰራተኛ 6. ሌላ (ይገለፅ) --- |  |  |
|  | የአባት የስራ ሁኔታ | | 1. የመንግስት ተቀጣሪ 4. የግል ስራ  2. የግል ተቀጣሪ 5. ገበሬ  3. የቀን ሰራተኛ 6. ሌላ ካለ ይገለጽ |  |  |
|  | ወርሃዊ የቤተሰብ ገቢ በብር | |  |  |  |
|  | ቋሚ የኪስ ገንዘብ ከቤተሰብሽ ታገኛለሽ | | 1. አዎ 2. የለም |  |  |
| የማህጸን እና ተያያዥ ጉዳዮች | | | | |  |
|  | የወር አበባ ማየት የጀመርሽበት እድሜ ስንት ነበር | |  |  |  |
|  | የወር አበባሽ ዑደት /6 ወር/ | | 1. የተስተካከለ 2. ያልተስተካከለ |  |  |
|  | የወር አበባሽ መጥቶ እስኪሄድ ያለዉ የቀን ብዛት | | 1. 2 ቀንና ከ2 ቀን በታች 3. ከ7 ቀን በላይ  2. ከ3 እስከ 7 ቀን |  |  |
|  | በወር አበባ ወቅት ህመም ይሰማሻል /በ3 ዑደት ውስጥ/ | | 1. አዎ 2. የለም |  |  |
| ስለወር አበባ ንፅህና አጠባበቅ ግንዛቤ በተመለከተ | | | | |  |
|  | የወር አበባ ከማየትሸ በፊት ስለ ወር አበባ ሰምተሸ ነበር? | | 1. ሰምቻለሁ 2. አልሰማሁም | መልሱ 2 ከሆነ ጥያቄ ቁጥር 19  ዝለሉ |  |
|  | ጥያቄ ቁጥር 18 መልስሽ ሰምቻለሁ ከሆነ ስለ ወር አበባ የስማሽዉ ከማን ነበር? | | 1. እናቴ 6. ጤና ባለሙያ  2. ትምህርት ቤት ውስጥ 7. ኢንተርኔት  3. ጓደኛ 8. አባት  4. ታላቅ እህት 9. ሌላ (ይገለፅ)---  5. ቴሌቪዥን |  |  |
|  | የወር አበባ ምንድነዉ ብለሽ ታስቢያለሽ? | | 1. የተፈጥሮ ዑደት 4. ሌላ ካለ (ይገለፅ)-  2. በበሽታ የሚመጣ 5. አላዉቅም  3. የፈጣሪ እርግማን |  |  |
|  | የወር አበባ መንስኤ ምንድን ነዉ ብለሽ ታስቢያለሽ? | | 1. ሆርሞን 4. ሌላ ካለ(ይገለፅ)  2. የፈጣሪ እርግማን 5.አላዉቅም  3. በበሽታ የሚመጣ |  |  |
|  | የወር አበባ ምንጭ ምንድን ነዉ? | | 1. ማኅፀን 4. ሆድ  2. ከረቤዛ(ብልት) 5. ሌላ ካለ (ይገለፅ)  3. የሽንት ፊኛ 6. አላዉቅም |  |  |
|  | ጤነኛ ልጃገረድ የወር አበባ በየስንት ቀኑ ታያለች ብለሽ ታስቢያለሽ? | | 1. ከ 21 ቀን በታች 4. አላውቅም  2. ከ 21 እስከ 35 ቀን  3. ከ 35 ቀን በላይ |  |  |
|  | ጤነኛ የወር አበባ አንዴ ከመጣ ለምን ያህል ጊዜ(ቀን) ይቆያል | | 1. ከ 2 ቀን በታች 4. አላዉቅም  2. ከ 3 እስከ 7 ቀን  3. ከ 7 ቀን በላይ |  |  |
|  | ስለ ወር አበባ ንፅህና ት/ቤት ተምረሽ ታዉቂያለሽ? | | 1. አዎ 2. የለም |  |  |
|  | የወር አበባ መጥፎ ጠረን አለው ብለሽ ታስቢያለሽ? | | 1. አዎ 2. የለም |  |  |
|  | የወር አበባ ንጹህ ያልሆነ ብለሽ ታስቢያለሽ? | | 1. አዎ 2. የለም |  |  |
|  | በወር አበባ ወቅት ንጽህናን አለመጠበቅ ለኢንፌክሽን ያጋልጣል ብለሽ ታስቢያለሽ? | | 1. አዎ 2. የለም |  |  |
|  | በወር አበባ ወቅት ንጽህናን መጠበቅ ከወር አበባ ጋር ተያይዞ የሚመጣ ህመምን ይቀንሳል ብለሽ ታስቢያለሽ? | | 1. አዎ 2. የለም |  |  |
|  | የወር አበባ ኡደት እድሜ ልክ ይኖራል ብለሽ ታስቢያለሽ? | | 1. አዎ 2. የለም |  |  |
| ስለወር አበባ ንፅህና የሚደረግ ዉይይትን በተመለከተ | | | | |  |
|  | | ስለ ወር አበባ ንፅህና ከጓደኞችሽ ጋር ትወያያለሽ | 1. አዎ 2. አልወያይም |  |  |
|  | | በቤተሰባችሁ ዉስጥ ስለ ወር አበባ በግልፅ ዉይይት ታደርጋላችሁ? | 1. አዎ 2. አናደርግም | 2 ከሆነ 33 ይዘለል |  |
|  | | ለተራ ቁ. 32 ጥያቄ መልስሽ አወ ከሆነ ስለ ወር አበባ በብዛት ከማን ጋር ነዉ የምትወያይዉ? | 1. ከእናቴ 4. ሌላ ካለ ይገለጽ…  2. ከአባቴ  3. ከታላቅ እህቴ |  |  |
|  | | በቤተሰባችሁ ዉስጥ ስለ ወር አበባ ውይይት ከሌለ ምክንያቱ ምንድነዉ? | 1. ነውር ስለሆነ 4.ሌላ ካለ ይገለጽ  2. ሚስጢር  3. ሁሉም |  |  |
|  | | ከመምህራኖችሽ ጋር ስለ ወር አበባ ትወያያለሽ? | 1. አዎ 2. አልወያይም |  |  |
| የትምህርት ቤቱን ዉሀና ንጽህና አሰጣጥ በተመለከተ | | | | |  |
|  | | በትምህርት ቤታችሁ በሳምንት ለምን ያህል ጊዜ ውሀ ይኖራል? ባለፈው 1 ወር | 1. 5 እስከ 7 ቀን 3. ከሁለት ቀን በታች  2. 2 እስከ 4 ቀን |  | |
|  | | መጸዳጃ ቤት መች መች መጠቀም ትችላላችሁ? | 1. በእረፍት ሰዓት ብቻ 2. በማንኛውም ሰዓት |  | |
| የወር አበባ ንፅህና አጠባበቅ በተመለከተ | | | | | |
|  | | በወር አበባ ወቅት የንጽህና መጠበቂያ/ሞዴስ/ ትጠቀሚያለሽ? | 1. አዎ 2. አልጠቀምም |  | |
|  | | ባለፉት 6 ወራት በወር አበባ ወቅት ምን አይነት የወር አበባ የንፅህና መጠበቂያ ተጠቅመሻል? | 1. ከሱቅ/ገበያ በሚሸጡ የንጽህና መጠበቂያ ሞዴሶች  2. ቤት ውስጥ በሚዘጋጅ የንጽህና መጠበቂያ  3. ሌላ ካለ (ይገለፅ)----------- |  | |
|  | | አጥበሽ መልሰሽ የምትጠቀሚዉን ልብስ/ፓድ/ በምን ታጥቢያለሽ? | 1 ሳሙናና ዉሃ  2. ዉሃ ብቻ 3. ሌላ ካለ (ይገለፅ)---- | የሚታጠብ ፓድ ለሚጠቀሙ | |
|  | | አጥበሽ መልሰሽ የምትጠቀሚዉን ልብስ/ፓድ/ የት ታደርቂያለሽ? | 1. የፀሀይ ብርህን በሚያገኘዉ ቦታ  2. የፀሀይ ብርህን በማይደርስበት  3. ሌላ ካለ (ይገለፅ)------ | የሚታጠብ ፓድ ለሚጠቀሙ | |
|  | | ማንኛውም የንፅህና መጠበቂያ የምትጠቀሚ ከሆነ በቀን  ምን ያህል ጊዜ የመቀያየር ልምድ አለሽ? | 1. አንድ ጊዜ 4. ከሶስት ጊዜ በላይ  2. ሁለት ጊዜ  3. ሶስት ጊዜ |  | |
|  | | በወር አበባ ወቅት ከረቤዛ(ብልት) የማፅዳት ልምድ አለሽ? | 1. አዎ 2. የለኝም |  | |
|  | | ለጥያቄ ቁ.43 አወ ከሆነ መልሱ በወር አበባ ወቅት ከረቤዛን(ብልት) ለማፅዳት ምን ትጠቀሚለሽ? | 1. ሳሙናና ዉሃ 4. ሌላ ካለ (ይገለፅ)  2. ዉሃ ብቻ  3. ሶፍት |  | |
|  | | በወር አበባ ወቅት በየቀኑ ገላሽን ትታጠቢያለሽ? | 1. አዎ 2. የለም |  | |
|  | | ለጥያቄ ቁ. 45 አወ ከሆነ መልሱ በወር አበባ ወቅት ገላሽን ለመታጠብ ምን ትጠቀሚያለሽ? | 1. ሳሙናና ዉሃ  2. ዉሃ ብቻ 3. ሌላ ካለ (ይገለፅ) ---- |  | |
|  | | የንፅህና መጠበቂያ (ፓድ) ከተጠቀምሽ በኃላ የት ታስወግጂዋለሽ | 1. ሜዳ ላይ 3. የቆሻሻ ማጠራቀሚያ  2. መፀዳጃ ቤት 4. ሌላ ካለ (ይገለፅ) |  | |
|  | | የተጠቀምሽበትን ፓድ በወረቀት ጠቅልለሽ ትጥያለሽ | 1. አዎ 2. አላደርግም |  | |
| ስለንጽህና መጠበቂያ ሞዴስ/ፓድ/ በተመለከተ | | | | | |
|  | | የንፅህና መጠበቂያ ሞዴስ የማትጠቀሚበት ምክንያት | 1. ዉደነት  2. በአቅራቢያየ ማግኘት ስለማልችል  3. ለማስወገድ አስቸጋሪ ስለሆነ  4. ዕዉቀቱ ስለሌለኝ  5. ስለማፍር  6. ሌላ ካለ (ይገለፅ)- | ከአንድ በላይ መልስ ይቻላል | |
|  | | ሞዴስ ለመግዛት ቤተሰብን ገንዘብ ትጠይቂያለሽ? | 1. አዎ 2. አልጠይቅም |  | |
|  | | ሞዴስ ለመግዛት ገንዘብ ከማን ትጠይቂያለሽ | 1. እናቴን 4. ወንድሜን  2. አባቴን 5. ሌላ ካለ ይገለጽ  3. እህቴን | ከአንድ በላይ መልስ ይቻላል | |
| የወር አበባ በሴቶች ትምህርት ላይ በተመለከተ | | | | | |
|  | | የወር አበባ ምክንያት በአማካይ ስንት ቀን ከትምህርት ቤት ቀርተሻል/ ባለፈው ሴሚስተር/ |  |  | |
|  | | በወር አበባ ምክንያት ከትምህርት ቤት የምትቀሪበት ምክንያት ምንድን ነበር? | 1. ልብስሽ ስለሚበላሽ  2. ስለምታፍሪ  3. ስለሚያምሽ  4. ምቾት ስለማይሰማሽ  5. ትምህርት ቤት መታጠቢያ ስለሌለ  6. ትምህርት ቤት ንጽህና መጠበቂያ ማስወገጃ ስለሌለ  7. ትምህርት ቤት ንጽህና መጠበቂያ ሞዴስ ስለሌለ  8. ትምህርት ቤት ንጽህና መጠበቂያ መቀየሪያ ክፍል ስለሌለ | ከአንድ በላይ  ምላሽ መስጠት ይቻላል | |
